# Structural and phenotypic analysis of Chikungunya virus *RNA* replication elements

**DOI:** 10.1093/nar/gkz640

**Published:** 2019-07-27

**Authors:** Catherine Kendall, Henna Khalid, Marietta Müller, Dominic H Banda, Alain Kohl, Andres Merits, Nicola J Stonehouse, Andrew Tuplin

**Affiliations:** 1 School of Molecular and Cellular Biology, Faculty of Biological Sciences and Astbury Centre for Structural and Molecular Biology, University of Leeds, Leeds, LS2 9JT, UK; 2 University of Ghent, Corneel Heymanslaan 10, B-9000 Ghent, Belgium; 3 MRC-Centre for Virus Research, University of Glasgow, Glasgow, G61 1QH, UK; 4 Institute of Technology, University of Tartu, Tartu 50411, Estonia

## Abstract

Chikungunya virus (CHIKV) is a re-emerging, pathogenic *Alphavirus* transmitted to humans by *Aedes* spp. mosquitoes. We have mapped the RNA structure of the 5′ region of the CHIKV genome using selective 2′-hydroxyl acylation analysed by primer extension (SHAPE) to investigate intramolecular base-pairing at single-nucleotide resolution. Taking a structure-led reverse genetic approach, in both infectious virus and sub-genomic replicon systems, we identified six RNA replication elements essential to efficient CHIKV genome replication - including novel elements, either not previously analysed in other alphaviruses or specific to CHIKV. Importantly, through a reverse genetic approach we demonstrate that the replication elements function within the positive-strand genomic copy of the virus genome, in predominantly structure-dependent mechanisms during efficient replication of the CHIKV genome. Comparative analysis in human and mosquito-derived cell lines reveal that a novel element within the 5′UTR is essential for efficient replication in both host systems, while those in the adjacent nsP1 encoding region are specific to either vertebrate or invertebrate host cells. In addition to furthering our knowledge of fundamental aspects of the molecular virology of this important human pathogen, we foresee that results from this study will be important for rational design of a genetically stable attenuated vaccine.

## INTRODUCTION

Chikungunya virus (CHIKV) is a member of the *Alphavirus* genus within the *Togaviridae* family and has become an increasingly important arbovirus in tropical and sub-tropical regions, responsible for a range of febrile and both acute and chronic arthralgic symptoms in humans. CHIKV is transmitted by *Aedes* spp. mosquitos - predominantly *Aedes aegypti* in tropical/sub-tropical regions but increasingly via *Aedes albopictus*, which has a wider geographical distribution, including across more temperate regions. Since ∼2000 CHIKV has undergone epidemic spread from beyond previously endemic areas of Africa and South-East Asia into India, China, Central and South America and the Caribbean (1,2). More sporadic outbreaks with confirmed autochthonous transmission have been recorded in Southern Europe and North America (3–5). Of increasing concern are recently identified genetic adaptations in the East Central South African (ECSA) strain of the virus, facilitating replication in the more widely distributed *Ae. albopictus* vector (6).

CHIKV is a small, enveloped, virus with a positive-sense RNA genome *∼*11.8 kb in length. The genome contains two open reading frames (ORF) flanked by 5′ and 3′ untranslated regions (UTRs) and separated by a non-coding intergenic region. The 5′UTR is 76nt in length and contains a 5′ type-0 N 7-methylguanosine cap for initiation of cap-dependent translation, the 3′UTR varies in length between ∼500 and ∼900nt and includes a 3′ polyadenylate tail. ORF-1 encodes the non-structural proteins nsP1–4, which form distinct modules of the viral replicase complex that is responsible for CHIKV RNA synthesis. Replication of genomic positive-sense RNA to full-length negative-sense intermediates occurs within membrane-bound replication complexes at the plasma membrane (7). As replication progresses, proteolytic processing of the non-structural protein precursors in the replicase complex, favours its association with the negative-strand and subsequent replication of positive-sense full-length genomic transcripts. Sub-genomic (26S) ORF-2 transcripts, encoding the structural proteins (capsid, envelope glycoproteins and 6K viroporin channel), are synthesized from a sub-genomic promoter in the negative strand (8).

Although replication of positive-sense RNA virus genomes initiates at the 3′ end of the molecule, 5′ RNA elements are often essential components of this process (9, 10). For example, RNA stem–loop SLA is located in the 5′UTR of flaviviruses, such as dengue virus, and is an essential promoter for initiation of genome replication (11). Following interaction between SLA and the viral RNA-dependent RNA polymerase (RdRp), long-range interactions between the 5′ and 3′ ends stabilize circularization of the virus genome and RdRp transfer to a 3′ promoter (12,13). Among other factors, 5′ promoters of genome replication play a role in the transition between translation and replication of RNA virus genomes and as a sampling mechanism, ensuring the integrity of the 5′UTR in each template (14).

Relatively little is known about the structure and function of RNA secondary or higher-order structures within the CHIKV genome or their potential function as RNA replication elements during virus replication. However, parallels may be drawn with other related alphaviruses, which have been studied in greater detail. A 51nt conserved sequence element (CSE), consisting of two short stem–loops within the nsP1 encoding region, has been predicted to be highly conserved across a range of alphaviruses and demonstrated by reverse-genetic analysis to play a role in the replication of Sindbis virus (SINV), Semliki Forest virus (SFV) and Venezuelan equine encephalitis virus (VEEV) (15–19). For SINV, disruption of the 51nt CSE was demonstrated to severely impair replication in mosquito and avian derived cells (15,20) with a lesser, yet still significant, effect on replication efficiency in mammalian derived BHK-21 cells (15,16). Mutation or deletion of the 51nt CSE in VEEV severely diminished or abolished RNA synthesis respectively in BHK-21 cells (21). However, a further study in VEEV indicated that either of the two stem–loops of the 51nt CSE may be deleted without serious consequence to replicative fitness in BHK-21 or mosquito derived cells, but deletion of both structures prevents productive VEEV infection (18). The 5′ terminal dinucleotide AU is highly conserved in alphaviruses including CHIKV (22) and is thought to be important in binding of the RdRp to the 3′ end of the negative-sense RNA during positive-strand synthesis. It has also been demonstrated that an RNA stem–loop structure at the 5′ terminus of the 5′UTR acts as an essential component in masking the alphavirus type 0 cap structure (21,23,24), which is otherwise recognised as non-self by host protein IFIT1 (25). During recent outbreaks, synonymous site variability within the nsP1-coding region of the CHIKV genome was shown to be restricted, indicating constraints on sequence variability, such as functional RNA elements, are likely in this region of the CHIKV genome (26). However, other than biochemical probing of the 51nt CSE (17), combined structural and reverse genetic analysis of RNA replication elements within this important human pathogen is lacking. Consequently, given the high level of sequence conservation in 5′ region, in addition to predicted RNA elements within the 5′ UTR and *nsp1* sequence of related alphaviruses, we chose to characterize the first 300nt of the CHIKV genome.

Here, we take a structure-led systematic reverse genetic approach, to investigate conserved RNA structures within the 5′UTR and adjacent nsP1 encoding region of CHIKV genome. We used biochemical SHAPE mapping of full-length genomic transcripts to provide an RNA structure map of the CHIKV 5′ ∼300nt region, at physiological body temperatures of *Aedes spp*. mosquitoes and humans. Using a systematic reverse genetic approach, we determine the importance of RNA structures, identified in the current study, during virus replication in human and mosquito derived cell lines and using sub-genomic replicon systems to investigate their roles in genome replication and translation. By combining structural mapping with such a systematic reverse genetic approach, we identify and dissect a number of novel RNA structures within the genomic transcript of CHIKV and demonstrate that they act as RNA replication elements that are essential for efficient replication of the virus genome, through both human/mosquito host dependent and independent mechanisms.

## MATERIALS AND METHODS

### RNA stem–loop nomenclature

Stem-loops were labelled according to the position of the 5′-most nucleotide of the structure, where nucleotide 1 represents the first nucleotide following the 5′ N 7-MeGTP cap of CHIKV ECSA (accession number DQ443544), e.g. SL85 begins 85 nucleotides from the cap. This standardized naming scheme facilitates reference to structures in coding or non-coding regions, is independent of higher-order interactions and can be logically extended or added to if additional structures are identified. Of particular relevance to this report, stem–loops designated here SL165 and SL194 correspond to the alphaviruses 51nt CSE observed previously in divergent alphaviruses (15,16,20,21,27).

### CHIKV cDNA plasmids and mutagenesis

The CHIKV infectious clone (ICRES) and sub-genomic replicons used in this study were derived from the LR2006_OPY1 La Réunion island isolate of the ECSA genotype (accession number DQ443544) (28). In the mono-luciferase sub-genomic replicon system (CHIKV_Rep) ORF-2 was replaced by a firefly luciferase gene, in the dual-luciferase replicons a Renilla luciferase encoding gene was additionally fused within nsP3 in ORF-1 (29). Sub-cloning of cDNA plasmid constructs, after mutagenesis, was carried out following XmaI and NotI double digest and agarose gel purification, ligation with T4 DNA ligase (NEB) and transformation into XL10-Gold Ultra-Competent cells (Agilent Technologies) according to the manufacturer's instructions. Mutagenesis was carried out using the Quik-Change II XL site-directed mutagenesis kit (Agilent Technologies) according to the manufacturer's instructions (mutagenesis primer sequences available on request). Plasmid cDNA was purified using GeneJET Plasmid Maxiprep kits (Thermo Fisher Scientific).

### *In vitro* RNA transcription

2 μg of *Not* I linearized cDNA plasmid, was used as template for the production of 5′ capped and uncapped RNA *in vitro* using SP6 mMessageMachine and MEGAscript kits, respectively (Thermo Fisher Scientific), according to the manufacturer's instructions. Following transcription, DNA template was removed by DNase 1 (Thermo Fisher Scientific) digestion and the RNA purified using RNeasy mini-kit columns (Qiagen). RNA integrity was confirmed by denaturing agarose gel electrophoresis and quantified by NanoDrop spectroscopy.

### Selective 2′hydroxyl acylation analysed by primer extension (SHAPE)

10 pmol of full-length CHIKV genomic RNA transcripts in 10 μl 0.5× Tris–EDTA (pH 8.0) (TE), was denatured at 95°C for 3 min, incubated for 3 min on ice before addition of 6 μl of folding buffer (330 mM HEPES (pH 8.0), 20 mM MgCl_2_ and 330 mM NaCl) and allowed to refold at either 37 or 28°C for 20 min. Samples were then divided into positive and negative reactions and incubated with either 1 μl of 100 mM *N*-methylisatoic anhydride (NMIA) (positive) or 1 μl of DMSO (negative) for 45 min at 37°C or 28°C. Each reaction was terminated by ethanol precipitation following the addition of 100 μl of EDTA (100 mM), 4 μl of NaCl (5 M) and 2 μl of glycogen (20 mg/ml). Following aspiration, samples were re-suspended in 10 μl 0.5× TE containing RNA secure (Thermo Fisher Scientific). For both the positive and negative reactions 5 μl of this full-length RNA was incubated with 1 μl of 10 μM 5′FAM labeled fluorescent oligonucleotide primer (AGACGGGCTACGCGTCACGC—ICRES nt position 318–337) (Sigma-Aldrich) and 6 μl ddH_2_O at 85°C for 1 min, 60°C for 10 min and 30°C for 10 min. A master mix of 4 μl superscript III reverse transcriptase buffer, 1 μl 100 mM DTT, 0.5 μl 100 mM dNTPs, 0.5 μl RNAseOUT, 1 μl ddH_2_O and 1 μl superscript III reverse transcriptase (Thermo Fisher Scientific) was added to each reaction, which were incubated for 30 min at 55°C—in order to reverse transcribe the 5′ 318 nucleotides for subsequent fragment size analysis.

For SHAPE sequencing ladder reactions, 6 pmol of *in vitro* transcribed RNA in 7.5 μl 0.5× TE buffer, 1 μl of 10 mM 5′HEX labelled oligonucleotide primer (Sigma-Aldrich) and 2 μl ddH_2_O was incubated at 85°C for 1 min, 60°C for 10 min and 30°C for 10 min. A master mix of 4 μl superscript III reverse transcriptase buffer, 1 μl 100 mM DTT, 0.5 μl 100 mM dNTPs, 0.5 μl RNAseOUT, 2 μl ddGTP and 1 μl superscript III reverse transcriptase was added before incubation for 30 min at 55°C.

Following incubation, all reverse transcription extensions were heated at 95°C for 3 min with 1 μl 4 M NaOH before cooling on ice with 2 μl 2 M HCl for 2 min. cDNA was precipitated in 4 μl 3 M NaAc, 4 μl 100 mM EDTA, 1 μl 20 mg/ml glycogen, and 60 μl 100% ethanol for 30 min at –80°C, pelleted by centrifugation, aspirated and resuspended in 40 μl deionized formamide. Samples were pooled with 20 μl of SHAPE sequencing ladder and stored at −80°C prior to fragment size analysis by capillary electrophoresis.

### SHAPE data analysis

Fragment size analysis of SHAPE extension products was conducted by capillary electrophoresis (DNA Sequencing and Services; part of the MRC-PPU Reagents and Services Facility, College of Life Sciences, University of Dundee, Scotland). SHAPE data was processed and normalized using the QuSHAPE software with default settings (30). As previously published, based on an average of at least three independent biological repeats, nucleotides with normalized SHAPE reactivities 0–0.3, 0.3–0.7 and >0.7 were taken to be unreactive, moderately reactive, and highly reactive respectively (30,31). *In silico* thermodynamic RNA structure and free energy predictions were carried out using UNAFOLD at 28 and 37°C (version 2.3) (32). Normalized SHAPE reactivates were used as constraints to generate a thermodynamic RNA structure model using the RNAstructure software (33,34). RNA structures were visualized and overlaid with normalized SHAPE reactivities using the VARNA software (35).

### Cell culture

Monolayers of the human hepatoma cell line Huh7 and Baby Hamster Kidney cell line BHK-21 were maintained in Dulbecco′s modified minimal essential medium (DMEM) supplemented with 10% (v/v) foetal bovine serum (Thermo Fisher Scientific), 0.1 mM non-essential amino acids, 2 mM l-glutamine and 100 U penicillin/100 μg streptomycin/ml (DMEM P/S). Cells were harvested using trypsin/EDTA, seeded at dilutions of 1:3 to 1:10 and maintained at 37°C in 5% CO_2_. *Ae. albopictus* derived cell line C6/36 was maintained in Leibovitz′s L-15 media supplemented with 10% (v/v) foetal bovine serum, 10% tryptose phosphate broth and 100 U penicillin/100 μg streptomycin/ml (Leibovitz's L-15/PS). C6/36 cells were passaged, following mechanical harvesting by scraping, at dilutions of 1:3 to 1:8 and maintained at 28°C without supplementing CO_2_.

### Virus production

1 × 10^6^ BHK-21 cells in 40 μl ice-cold DEPC-PBS were electroporated with 2 μg 5′-capped *in vitro* transcribed RNA in a 4 mm electrocuvette, with a single square wave pulse at 260 V for 25 ms using a Bio-Rad electroporator, before seeding into a T75 flask in 10ml DMEM P/S. After 24 h, supernatant was aspirated and titred by plaque assay.

### Virus infections

Huh7 cells and C6/36 cells were seeded in 24-well plates at 1 × 10^5^ cells/well. After 24 h, monolayers were washed with PBS and infected with CHIKV at a MOI of 1 (calculated based on titre in BHK21 cells) in 200 μl of serum and P/S free media. One-hour post-infection, monolayers were washed with PBS and maintained for 24 h in complete media as described previously, following which supernatant was aspirated, clarified and titred by plaque assay.

### Plaque assay virus titration

BHK-21 cells were seeded in a 6 well plate at 4 × 10^6^ cells/well and maintained in DMEM P/S as previously described. The following day, monolayers were washed with PBS and infected with 200 μl of 10-fold serial dilutions of CHIKV infection supernatant and incubated at 37°C. One-hour post-infection, monolayers were washed with PBS and covered with a 0.8% methylcellulose DMEM P/S overlay. Following a 48-h incubation, monolayers were fixed and stained (5% paraformaldehyde and 0.25% crystal violet respectively) before plaques were counted and virus titres expressed in plaque-forming units per ml (PFU/ml).

### Strand-specific quantification of CHIKV RNA

Huh7 and C6/36 cells were infected with CHIKV as described above, with two modifications: 12-well plates were used and seeding density was increased to 6 × 10^5^ cells/well for C6/36. At 24 hpi, total RNA was extracted from cells using TRI Reagent^®^ Solution (Applied Biosystems) according to the manufacturer's protocol. The strand-specific qPCR (ssqPCR) was performed according to the protocol described by Plaskon and colleagues (36). Briefly, 500 ng of RNA were reverse-transcribed with gene specific primers using the SCRIPT cDNA Synthesis Kit (Jena Bioscience) according to the manufacturer's instructions. 100 ng of strand-specific cDNA was used as template for the quantitative PCR, performed with the qPCRBIO SyGreen Blue Mix Lo-ROX (PCR Biosystems) with gene specific primers (Supplementary Table S1) amplifying a 94 bp region of the CHIKV nsP1 encoding sequence using the following PCR program: 95°C for 2 min, 40× (95°C for 5 s, 60°C for 30 s), dissociation curve 60–95°C as pre-defined by the Mx3005P thermal cycler (Agilent technologies). *In vitro* transcribed CHIKV ICRES RNA was reverse transcribed and a cDNA dilution series employed as a standard to quantify copy numbers in the respective samples. All experiments were performed for a minimum of three independent repeats.

### Sub-genomic replicon transfection and analysis

Cells were seeded in 24-well plates at 5 × 10^4^ cells/well (Huh7) and 1 × 10^5^ (C6/36) and maintained overnight in DMEM/PS or Leibovitz's L-15/PS respectively—before monolayers were transfected using Lipofectamine 2000 transfection reagent (Thermo Fisher Scientific), following the manufacturers protocol. Briefly, monolayers at ∼80% confluence were washed twice in PBS and 500 μl of Opti-MEM reduced serum media (Thermo Fisher Scientific) before 100 μl of transfection medium was added in a drop-wise manner. Transfection medium was prepared with 2 μl Lipofectamine 2000 and 500 ng of capped sub-genomic replicon RNA, made up to 100 μl with Opti-MEM media. Following transfection monolayers were maintained for 6 hours before washing twice with PBS, lysed with 0.1 ml passive lysis buffer (Promega) and stored at −80°C prior to analysis using luciferase assay reagent (Promega) and a FluoStar Optima luminometer to measure levels of luciferase expression, which was then expressed as Relative Light Units (RLU). For later time points, monolayers were washed twice with PBS at 6 h post transfection and maintained under previously described growth conditions before harvesting and analyses as described earlier.

### Statistical analysis

Statistical analysis was carried out using two-tailed Student's *t*-tests for unpaired samples of equal variance. *P* values of ≤0.05 (*), ≤0.01 (**), ≤0.001 (***) were used to represent degrees of significance for each mutant compared to wild-type. Each experiment was repeated to gain a minimum of three independent biological repeats.

## RESULTS

### SHAPE mapping of the 5′UTR and adjacent nsP1 encoding region

**Figure 1.**
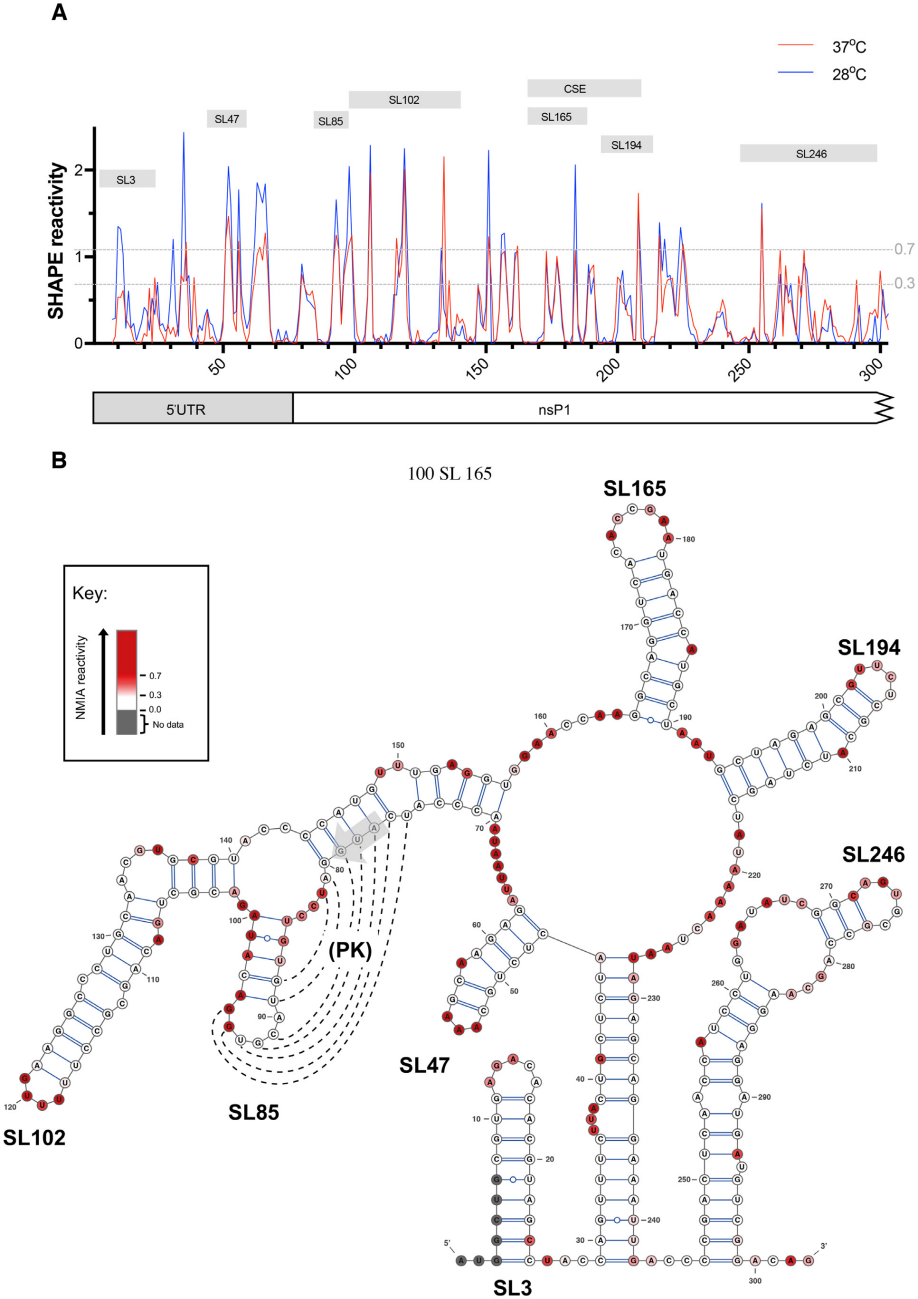
(**A**) Relative SHAPE reactivity for CHIKV 5′ UTR and adjacent nsP1 encoding region at 37°C (red) and 28°C (blue) (*n* = 3). Nucleotides with normalized SHAPE reactivities of 0–0.3, 0.3–0.7 and >0.7 were taken to be unreactive (base-paired), moderately reactive (intermediate levels of base pairing), and highly reactive (unpaired) respectively. Predicted stem–loop positions and conserved sequence element (CSE) annotated as labelled grey boxes. (**B**) 37°C SHAPE reactivities for individual nucleotides overlaid onto a 37°C thermodynamically derived model of RNA folding, generated using SHAPE-directed constraints. The AUG start codon of nsP1 is denoted by a grey arrow. SHAPE reactivities are shown as a heat map: grey indicates no data, white SHAPE reactivities between 0–0.3 and increasing intensities from light pink to dark red indicate increasing SHAPE reactivities, as denoted by the key. High reactivity (red) denotes unpaired nucleotides whereas low reactivity (white) denotes base-paired nucleotides. Predicted stem–loops are labelled SL3, SL47, SL85, SL102, SL165, SL194 and SL246. PK denotes a putative pseudoknot structure, where dotted lines represent potential base-pairing.

We investigated the RNA structure of the 5′UTR and adjacent nsP1 encoding region by SHAPE analysis of nucleotides 1–318 of full-length *in vitro* transcribed CHIKV RNA transcripts, derived from the ICRES cDNA template, comparing nucleotide reactivities following folding of RNA transcripts at 37 and 28°C (human and *Ae. albopictus* cell permissive temperatures respectively). Although, some differences in normalized levels of reactivity were observed for individual nucleotides, overall the positions of both increased and supressed SHAPE reactivity exhibited an extremely high degree of convergence (Figure 1A). Similarities in NMIA reactivities implied that RNA structures within this region were not fundamentally influenced by differences in human and mosquito host cell permissive temperatures. These results were in concordance with SHAPE-constrained *in silico* thermodynamic folding predictions, which again predicted that RNA structure within this region of the virus genome was independent of differences in permissive temperature between vertebrate and invertebrate hosts (Figure 1B and Supplementary Figure S1). Given, the close correlation in SHAPE reactivity profiles between the two permissive temperatures, results at 37°C were used for structure-based design of RNA stem–loop mutants for reverse genetic analysis.

SHAPE-constrained thermodynamic folding predicted seven discreet stem–loop structures – two within the 5′UTR (SL3 and SL47) and five within the adjacent nsP1 encoding region of ORF1 (SL85, SL102, SL165, SL194 and SL246). SL3 has previously been mapped in CHIKV and related alphaviruses and demonstrated to function in viral immune evasion, mimicking the methylated cap structure and avoiding recognition by IFIT-1 (25), consequently it was not investigated further in this study. Whilst SL165 and SL194 correspond to the 51nt nsP1 CSE, S47, SL85, SL102 and SL246 are novel structures and their potential functions have not previously been investigated.

For six of the seven structures (SL3, SL47, SL102, SL165, SL194 and SL246) there was a very high degree of concordance between the structural predication and NMIA reactivity. Interestingly however, this was not the case for SL85 - located directly down-stream of the AUG start codon. SHAPE reactivities within SL85 indicated a high degree of exposure for nucleotides of the stem and low-reactivity within nucleotides of the predicted terminal loop. The terminal loop of SL85 was observed to be complementary to a region of down-stream sequence, overlapping the AUG start codon. Formation of a higher order pseudoknot interaction between these complementary sequence domains may be consistent with the SHAPE reactivity profiles observed, while contradiction between the SHAPE and *in silico* structure predictions may suggest that SL85 and its adjacent sequence are involved in dynamic secondary and higher-order RNA-RNA interactions (Figure 1B).

### Phenotypic consequences of stem–loop mutagenesis

In order to validate the phenotypic consequences of disrupting predicted RNA stem–loops, mapped earlier in the study by SHAPE, we took a reverse genetic approach. Initially individual stem–loops (SL47, SL85, SL102, SL165, SL194 and SL246) were disrupted by synonymous site mutagenesis and resulting phenotypic changes, to different stages of the virus replication cycle, assayed in human and mosquito derived cell lines. A systematic and rational approach was taken to mutagenesis design, in which synonymous substitutions were designed to disrupt the predicted base-paired duplex stems of individual mutants in such a way that further synonymous mutations could then be incorporated to restore predicted base-pairing. The intention of this approach was that by phenotypic comparison of a stem–loop mutant with disrupted duplex stem, to one in which base-pairing was restored, we would be able to distinguish between phenotypic changes due to disruption in stem–loop structure and those due to alterations in primary synonymous nucleotide sequence. Furthermore, as compensatory mutations were designed based on a structure model of the positive genomic copy of the CHIKV genome, rescue of wild-type phenotype by this approach would confirm the importance of a stem–loop in the positive-sense genomic strand - rather than due to unforeseen disruption of potential functional elements in the negative strand intermediate of the viral genome.

**Figure 2.**
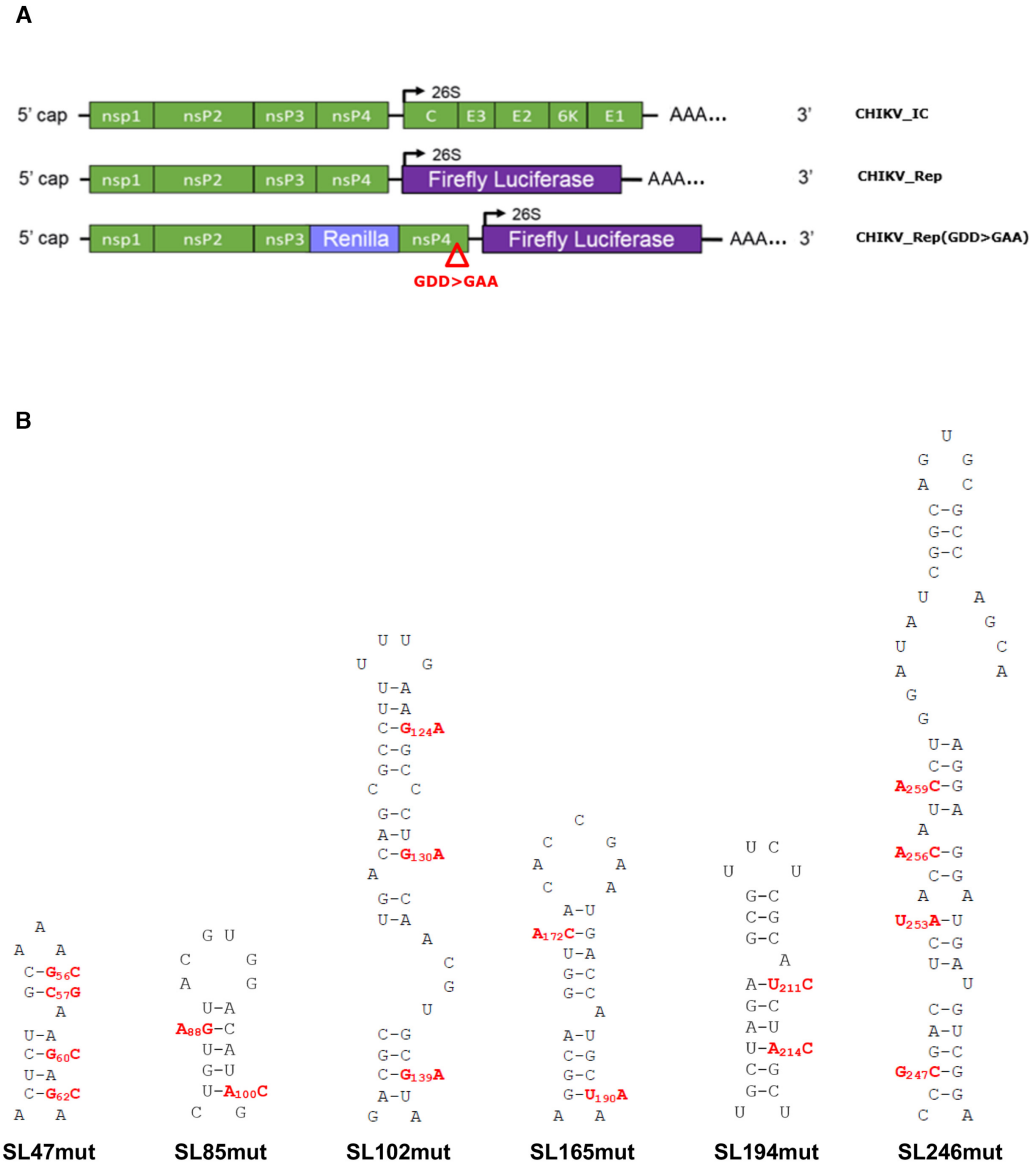
(**A**) Schematic representation of CHIKV infectious clone (CHIKV_IC) and sub-genomic replicon constructs—reporting genome replication through expression of Firefly luciferase (CHIKV_Rep) or translation through expression of Renilla luciferase, in the context of a replication deficient RdRp GDD>GAA mutant (indicated by red triangle) (CHIKV_Rep(GDD>GAA)). Non-structural proteins nsp1–4 are encoded by the first ORF. Structural proteins C, E1–3 and 6K are translated from a sub-genomic RNA, termed 26S RNA (black arrow), encoded by the second ORF. (**B**) Schematic representation of individual RNA stem–loops and associated mutations (red) designed to destabilize the base-pairing of the heteroduplex stem. All mutations in the nsp1 encoding region are synonymous.

Stem-loop mutants were incorporated into the full-length CHIKV infectious clone (CHIKV_IC) and corresponding CHIKV sub-genomic replicon systems (Figure 2A). The outcome of CHIKV genome replication events was measured using a sub-genomic replicon, which encodes viral non-structural proteins nsP1-nsP4, while ORF-2 was replaced with a Firefly luciferase reporter gene (CHIKV_Rep). ORF-1 translation phenotypes were measured using a replication deficient dual luciferase replicon (CHIKV_Rep(GDD>GAA)), in which an additional reporter gene (*Renilla* luciferase) was incorporated into ORF-1, as a fusion within the gene encoding nsP3. CHIKV_Rep(GDD>GAA) undergoes initial translation to express the reporter in ORF-1 but subsequent transcription events cannot occur, allowing translation of the non-structural proteins to be studied in isolation from genome replication.

### Individual stem–loops function as RNA replication elements

**Figure 3.**
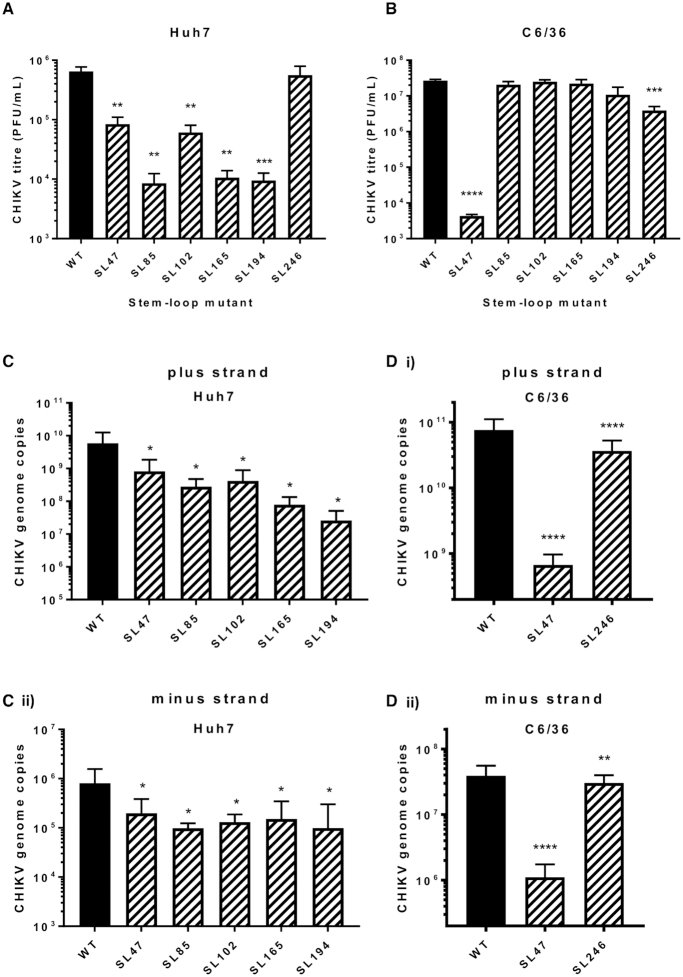
Replication phenotype of infectious wild type (WT) CHIKV (black bar) compared to virus bearing mutations predicted to destabilize the heteroduplex stem RNA structures (hatched bars), in (**A**) Huh7 human and (**B**) C6/36 *Ae. albopictus* cells. Viral genome copy number following CHIKV infection in (**C**) Huh7 and (**D**) C6/36 cells for 24 h for WT and destabilized mutants. Data is shown for (**i**) positive strands and (**ii**) negative strands of the CHIKV genome.

The phenotypic consequences for CHIKV replication, of individually destabilising base pairing within the duplex stems of SL47, SL85, SL102, SL165, SL194 and SL246 (Figure 2B), was compared between mutant and wild-type viruses in both human (Huh7) and mosquito derived (C6/36 – *Ae. albopictus*) host cell systems. Released virus was collected at 24 hours post infection with an equal MOI and titred by plaque assay (Figure 3). Interestingly SL47, located within the 5′UTR, was the only structure to significantly impact viral replication in both human and mosquito host cells. Compared to wild-type CHIKV, disruption of SL47 significantly inhibited virus replication by ∼1 log in Huh7 cells and ∼4 logs in C6/36 cells. The structures within the nsP1-encoding region had a more host specific effect. Disruption of SL85, SL102, SL165 and SL194 significantly inhibited CHIKV replication by between >1 and >2 logs compared to wild-type in Huh7 cells, while having no effect on virus replication in C6/36 cells. In contrast, disruption of SL246 significantly inhibited CHIKV replication by ∼1 log in C6/36 cells, yet replication in Huh7 cells was not affected. Huh7 cell-type specific phenotypes were confirmed in Huh7 cells cultured and infected at 28°C (i.e. the lower C6/36 permissive temperature). When grown at this lower temperature, disruption of SL102, SL165 and SL194 significantly inhibited virus replication, to a similar degree as observed at 37°C (Supplementary Figure S2). These data therefore confirm Huh7 cell-type specificity, rather than the lower permissive temperature (28°C) of C636 cells stabilizing the mutated stem–loops. Disruption of SL85 also resulted in significant inhibition of virus replication in Huh7 cells, when grown at 28°C. However, replication of the SL85 mutant was significantly less impaired than at 37°C - suggesting that, while SL85 functions in a host-cell dependent manner, the lower permissive temperature of C636 cells may also have had a stabilising effect on the mutated stem–loop.

For mutants exhibiting significantly impaired replication in the virus release assay, we quantified CHIKV genomic and intermediate minus-strand RNA copy number in Huh7 and C636 cells by ssqPCR. Consistent with previous results, significantly reduced levels of both genomic positive-strand and intermediate minus-strand RNA compared to wild-type were observed in Huh7 cells for SL47, SL85, SL102, SL165 and SL194 (Figure 3C). Similarly, levels of both RNA species were significantly reduced compared to wild-type for mutants SL47 and SL246 in C636 cells (Figure 3D).

### Individual RNA replication elements function during virus genome replication

**Figure 4.**
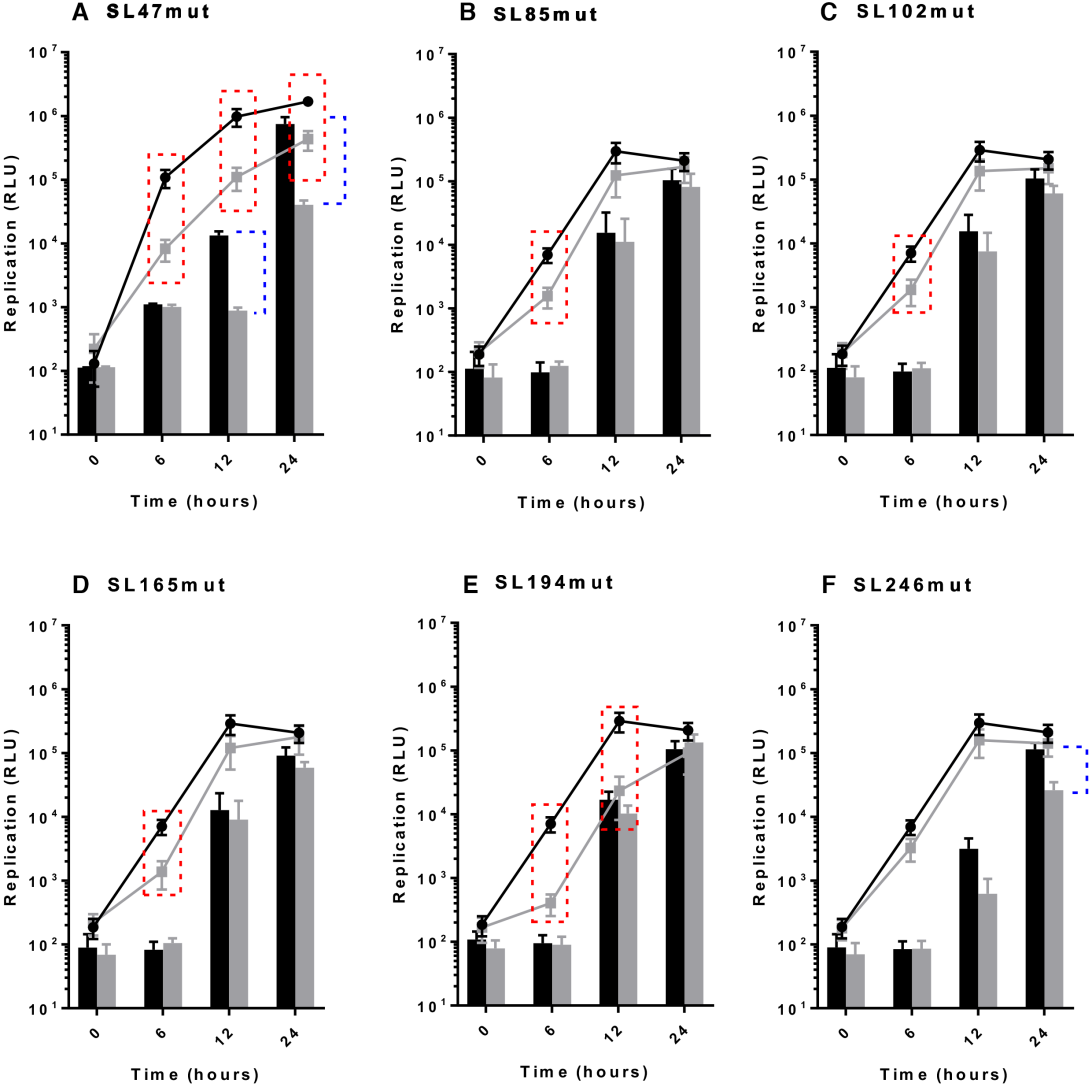
Replication phenotype of sub-genomic CHIKV replicons in Huh7 human (line graph) and C6/36 Ae. albopictus (filled bars) cells. Replicons with WT RNA structure (black) compared to replicons bearing mutations predicted to destabilize the heteroduplex stem of RNA structures (grey) (**A–F**). Significance depicted by dotted lines for each mutant compared to WT in Huh7 human (red boxes, *P*<0.05) and C6/36 mosquito (blue brackets, *P*<0.05) cell lines. For example, replicon of WT RNA structure replicates significantly better than SL85mut replicon in Huh7 cells at 6 hours, but there is no significant inhibition of replication in C6/36 cells.

Following the results of infection studies, whereby disruption of individual RNA structures in the genome were shown to inhibit CHIKV replication, we went on to investigate at what stage of the viral lifecycle they function. In order to examine potential roles during genome replication - in isolation from other stages of the replication cycle, such as packaging, effects of the stem–loop mutations on replication of a CHIKV sub-genomic replicon system were measured over time (Figure 4). Results from these sub-genomic replicon studies recapitulated the stem–loop mutant phenotypes, observed in the infectious virus assays. Compared to wild-type, disruption of SL47 significantly inhibited replication in both Huh7 and C6/36 cells (Figure 4A), while disruption of SL85, SL102, SL165 and SL194 significantly inhibited replication in Huh7 cells and had no effect in C636 cells (Figure 4B–E). Likewise, also in agreement with the infectious virus study, disruption of SL246 significantly inhibited sub-genomic replicon replication in C6/36 cells and had no significant effect in Huh7 cells (Figure 4F).

**Figure 5.**
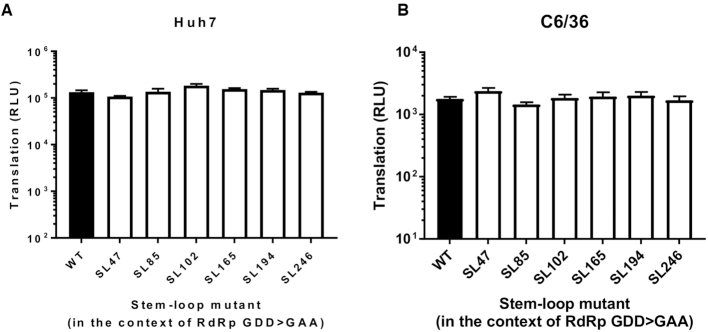
Translation phenotype in the context of replication-deficient sub-genomic CHIKV replicon system (CHIKV_Rep(GDD>GAA)) in (**A**) Huh7 and (**B**) C6/36 cells at 6 h post-transfection for Huh7 cells and 8 h post-transfection for C6/36 cells. Replicons with WT RNA structure (black) are compared to replicons bearing mutations predicted to destabilize the heteroduplex stem of RNA structures (white).

Whilst clearly demonstrating that the stem–loops function during genome replication of the virus, the sub-genomic replicon studies do not distinguish between different stage of this process, such as initiation of transcription or translation of the ORF-1 non-structural polyprotein. Mutations inhibiting translation of ORF-1 would impair efficient production of replicase complexes, thereby inhibiting the down-stream process of genome replication. In order to investigate the possibility that disruption of the stem–loops inhibited ORF-1 translation, we incorporated the stem–loop mutations into a replication-incompetent sub-genomic replicon (CHIKV_Rep(GDD>GAA)), in which ORF-1 translation from input RNA could be measured by expression of a *Renilla* luciferase reporter fused within nsP3, (Figure 2A). Such an approach enabled the efficiency of ORF-1 translation to be measured and compared between wild-type and stem–loop mutant sub-genomic transcripts, in isolation of genome replication. Translation of input sub-genomic replicon 5′capped transcripts was measured at 6 hours and 8 hours post-transfection into Huh7 and C6/36 cell lines respectively (Figure 5). No significant differences in levels of translation were observed between the CHIKV_Rep(GDD>GAA) replication deficient mutant encoding wild-type stem–loops to those encoding the mutant structures. In combination with our earlier results, from replication competent sub-genomic replicon mutants, these results indicate that the individual stem–loops do not influence ORF-1 translation but rather function at the level of genome replication.

### Stem-loops enhance CHIKV replication in a structure-dependent manner

**Figure 6.**
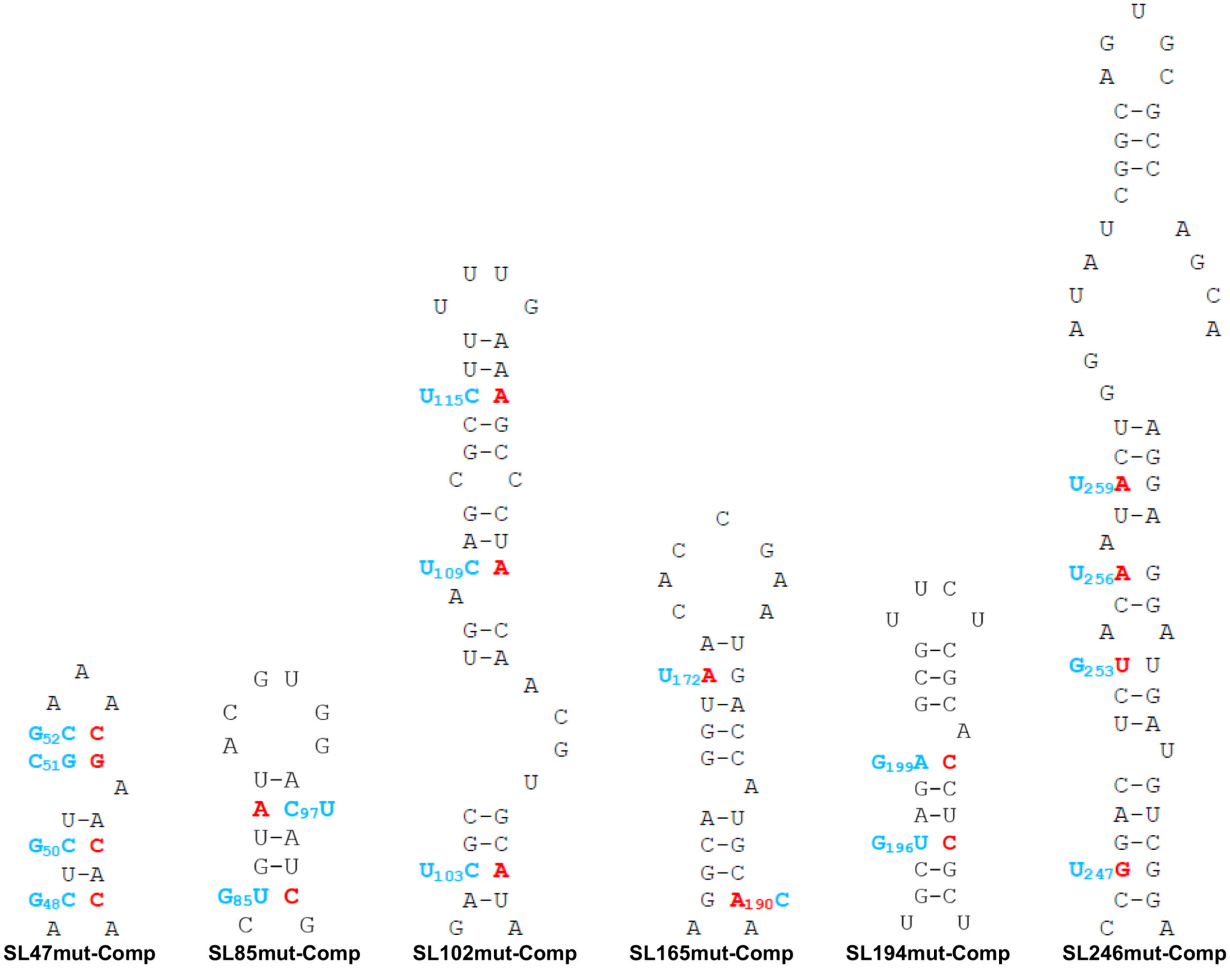
Mutations predicted to destabilize the heteroduplex stem of CHIKV genomic RNA structures (red) and compensatory mutations predicted to restore base-pairing (blue). All mutations in the nsp1 encoding region are synonymous.

**Figure 7.**
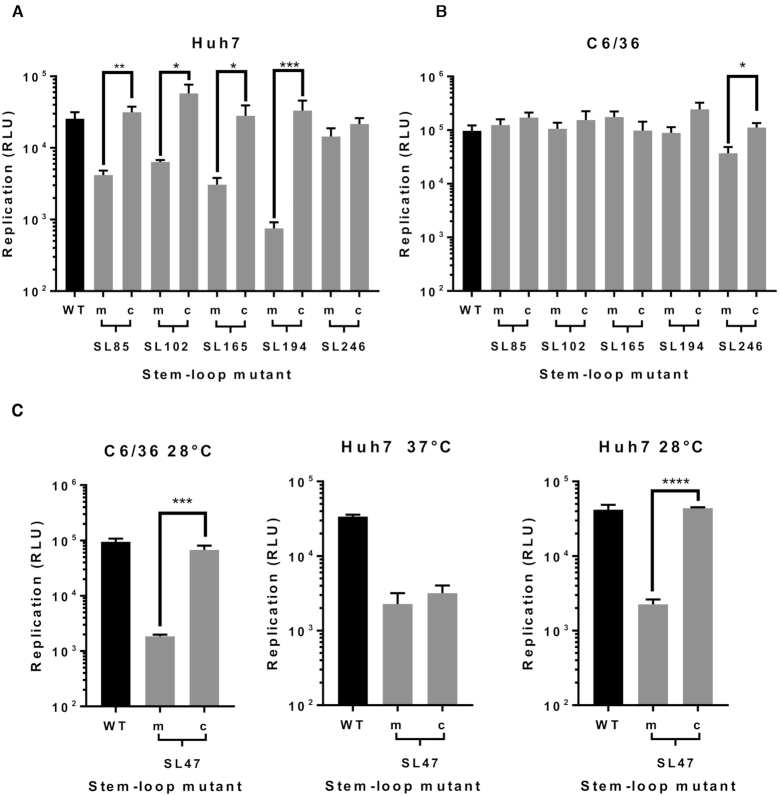
Replication phenotype of sub-genomic CHIKV replicons in (**A**) Huh7 and (**B**) C6/36 cells, after 6 and 24 h respectively. Replicons with WT RNA structure (black) are compared to replicons bearing mutations (grey) predicted to destabilize (m) or restore (c) the heteroduplex stem of individual genomic RNA structures. (**C**) Replication phenotypes for SL47mut (m) and SL47mut-Comp (c) shown in both cell types, alongside data from Huh7 cells at 28°C.

**Figure 8.**
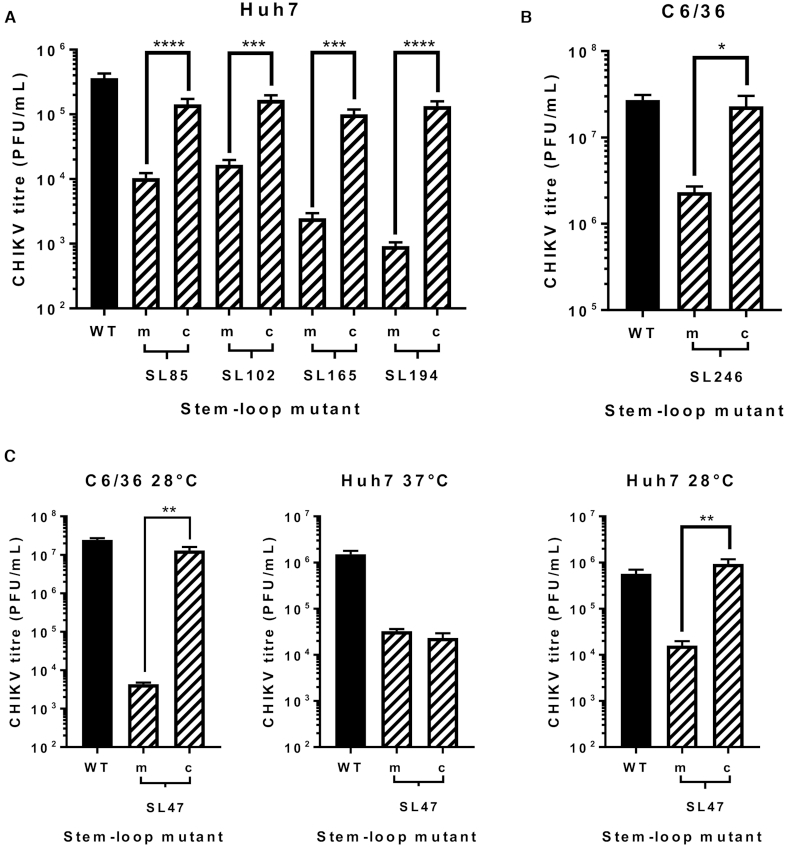
Replication phenotype of WT CHIKV (black) compared to virus containing mutations (hatched bars) predicted to destabilize (m) or restore (c) the heteroduplex stem of genomic RNA structures. Results are shown only for cell lines in which destabilisation of stems exerts an effect on replication: (**A**) Huh7, (**B**) C6/36 cells and (**C**) both cell types. Replication phenotypes for SL47mut (m) and SL47mut-Comp (c) shown in both cell types, alongside data from Huh7 cells at 28°C.

In order to confirm that observed mutant phenotypes were due to synonymous-site disruption of base-pairing within predicted stem–loops (Figure 2B), rather than due to alteration of the primary nucleotide sequence, or off target disruption of RNA elements in the complementary negative-strand, we incorporated further compensatory synonymous mutations; designed to restore base-pairing within the duplex-stem of each predicted structure, without reverting to wild-type nucleotide sequence (Figure 6). In line with our hypothesis, we analysed the ability of the compensatory mutations to rescue wild-type levels of replication in both the sub-genomic replicon and infectious virus systems in Huh7 and C6/36 cells (Figures 7 and 8 respectively).

For each stem–loop within the nsP1 encoding region, restoration of base-pairing by compensatory synonymous-site substitutions restored wild-type levels of sub-genomic replicon replication in the relevant host cell line (Figure 7A and B). Similarly, restoration of SL85, SL102, SL165 and SL194 significantly rescued CHIKV virus replication compared to the disrupted mutants in Huh7 cells and likewise SL246 was restored to wild-type replication levels in C6/36 cells (Figure 8A and B). These results are consistent with observed suppression of replication due to disruption of SL85, SL102, SL165 and SL194 structure, rather than alterations in the primary nucleotide sequence or off target disruption of RNA structure elements in the negative-strand replication intermediate.

As inconsistencies in the SHAPE reactivity profile of SL85 suggested that this region may be structurally dynamic, we made further substitutions to destabilise and subsequently restore base-pairing to a potential pseudoknot interaction within this region (Supplementary Figure S3A). The upstream region of this potential interaction overlaps the ORF-1 start-codon and the down-stream side is integral to SL85, as such the range of potential synonymous nucleotides available for mutagenesis was extremely limited. While mutagenesis at the upstream side inhibited CHIKV replication, no effect was observed following mutagenesis of the down-stream side and wild-type levels of replication were not rescued by compensatory substitutions (Supplementary Figure S3B). Consequently, given the limited range of mutagenesis options and the potential for disrupting the start codon Kozak consensus, the structure and functionality of alternative interactions within this region remains unclear.

Within the 5′UTR, restoration of base-pairing in the duplex stem of SL47 restored wild-type levels of CHIKV replication in mosquito derived C6/36 cells at the permissive temperature of 28°C, for both the sub-genomic replicon (Figure 7C) and infectious virus (Figure 8C). Although, compensation of wild-type phenotype was not observed in human derived Huh7 cells at 37°C, full rescue in these cells did occur when they were maintained at 28°C for the duration of the assay. Temperature-dependent rescue of wild-type phenotype in *SL47mutComp* is consistent with SL47 *in silico* UNAFOLD predicted folding free energies at 37°C (wild-type −2.9 kcal/mol and *SL47mutComp* −2.3 kcal/mol), indicating that *SL47mutComp* is less thermodynamically stable than the wild-type but that this could be compensated for by increased folding free energies at 28°C (wild-type −4.35 kcal/mol and *SL47mutComp* −3.75 kcal/mol).

### Phenotypic consequences of substitutions within stem–loop single-stranded regions

**Figure 9.**
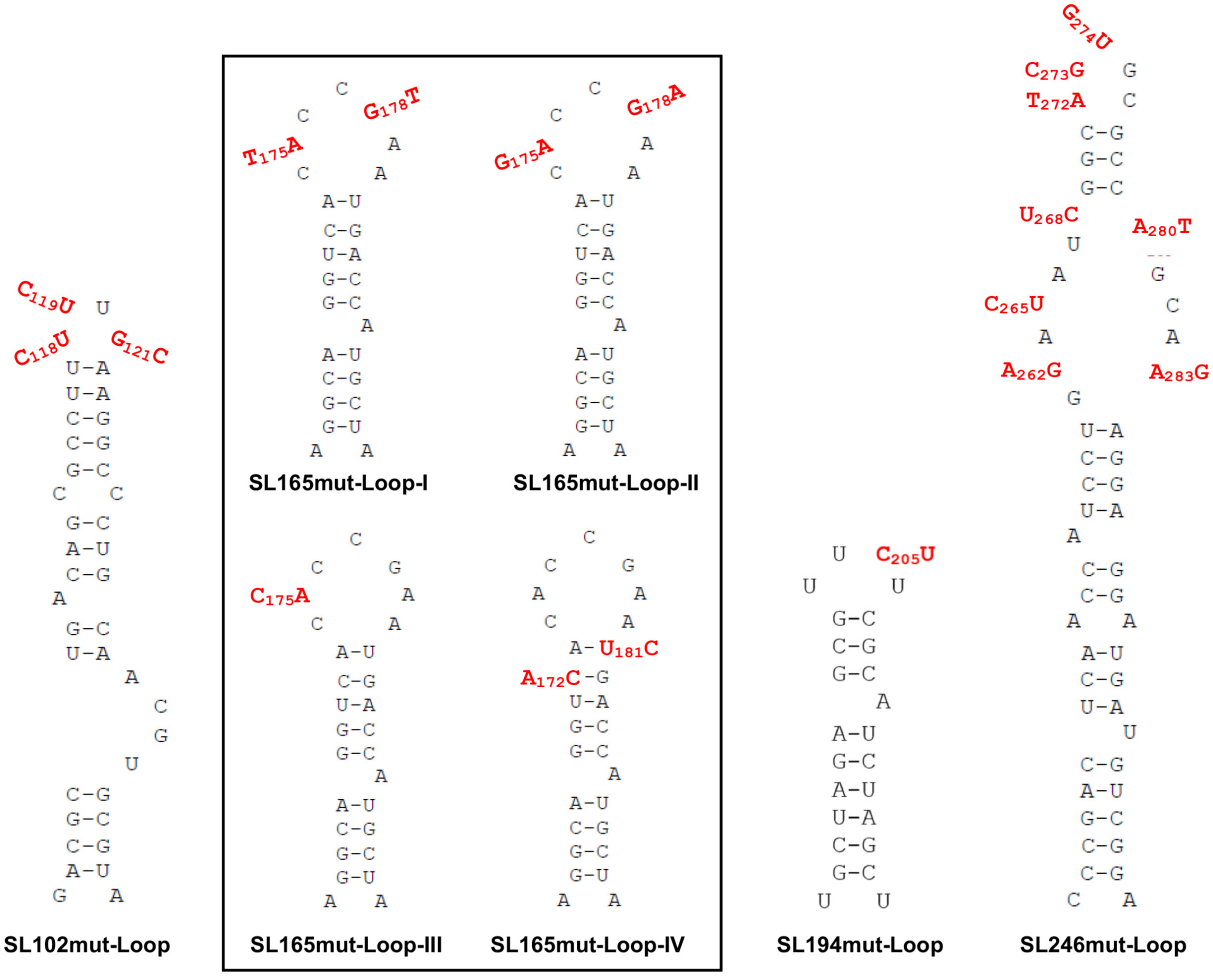
Substitutions (red) in single-stranded regions of CHIKV genomic RNA structures. Black box indicates several distinct loop mutants in SL165. Mutations are synonymous in SL165 and SL194. Mutations are more extensive and non-synonymous in SL102 and SL246.

**Figure 10.**
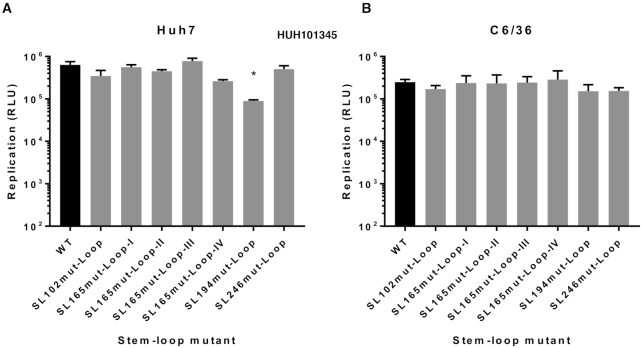
Replication phenotype of sub-genomic CHIKV replicons with WT RNA structure (black) compared to replicons bearing mutations (grey) in terminal loop and single-stranded bulge regions of RNA structures in (**A**) Huh7 and (**B**) C6/36 cells.

**Figure 11.**
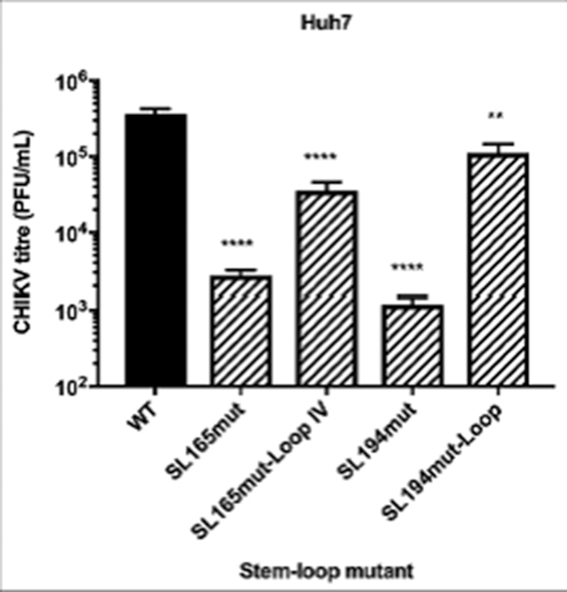
Replication phenotype of WT CHIKV (black) compared to viruses containing mutations (hatched bars) in terminal loop and single-stranded bulge regions of genomic RNA structures in Huh7 cells.

In order to investigate the role of single-stranded regions within the nsP1 region stem–loops, synonymous substitutions were introduced into a number of terminal-loop and bulge regions of the RNA replication elements, that had been predicted in our SHAPE studies to be unpaired (Figure 9). Replication phenotypes, compared to wild-type, were measured in the sub-genomic replicon system in both Huh7 and C6/36 cells (Figure 10). Levels of replication for mutants with substitutions in the single stranded regions of SL102, SL165 and SL246 were indistinguishable from wild-type in Huh7 and C6/36 cells—suggesting that the stem–loop structures themselves, rather than the primary sequence of unpaired motifs is important for efficient CHIKV replication. We did observe that increasing the number of unpaired nucleotides in the terminal-loop of SL165, by substitutions predicted to disrupt base-pairing in the apex of the duplex stem (SL165mut-Loop-IV), resulted in small reduction in sub-genomic replicon (Figure 10A) and virus (Figure 11) replication. However, inhibition of replication compared to wild-type was only significant in the virus system and disrupting base-pairing in the duplex stem could not be excluded as a contributing factor.

The only substitution in a single-stranded region of the replication elements, which was observed to inhibit replication, was a synonymous C>U substitution within the terminal loop of SL194 (SL194mut-Loop), reproducing a UUUU sequence motif that is conserved at this locus in divergent alphaviruses—such as SINV, SFV and VEEV. This substitution significantly inhibited replication in Huh7 cells by ∼1 log compared to wild-type, in both the sub-genomic (Figure 10A) and infectious virus (Figure 11) systems, indicating that the primary terminal loop sequence of SL194, in addition to requirement for an intact duplex stem, is important for its function as an RNA replication element during CHIKV genome replication.

## DISCUSSION

Here we present the first systematic reverse-genetic analysis of RNA structural elements within the 5′UTR and adjacent nsP1 encoding regions of the CHIKV genome, informed by biochemical SHAPE mapping of full-length viral genomic RNA transcripts at physiological temperatures relevant to both human and mosquito host cells. We describe novel RNA structural elements, within the positive-strand genomic copy of the CHIKV genome, that function in host independent and dependent mechanisms (i.e. in either human or mosquito derived host cells or in both) during efficient replication of the virus genome.

As an arbovirus, CHIKV must maintain fitness in both vertebrate and invertebrate host-cell environments. In order to investigate how the different permissive temperature conditions of mosquito and human cells may influence the thermodynamic stability and resulting folding structure of the CHIKV genome, we compared SHAPE mapping of full-length CHIKV genomic transcripts at mosquito and human host-cell permissive temperatures (28 and 37°C respectively). Although, some differences in relative levels of SHAPE reactivity were observed between the two thermodynamic folding conditions, their overall reactivity profiles were remarkably similar (Figure 1 and Supplementary Figure S1). In particular, SHAPE reactivity profiles were strikingly similar within the predicted stem–loop structures, indicating that different ambient growth temperatures are not likely to result in alternative folding structures for essential replication elements in this region of the CHIKV genome. This suggests that host specificity of RNA replication elements identified and analysed in this study is more likely due to interaction with host-specific *trans*-activating factors, than from different invertebrate and vertebrate host-cell temperatures stabilizing alternative RNA structure conformations.

Results presented here indicate that the RNA structures mapped in this study within the nsP1 encoding region are required for efficient CHIKV genome replication in a host-dependent manner, while SL47 in the 5′UTR acts during genome replication in both human and mosquito host cells – presumably via either a mechanism shared by the two hosts or through two distinct pathways. To our knowledge, SL47 represents a novel, previously unreported, RNA replication element. SL47 is highly conserved across CHIKV genotypes and closely related viruses such as O’nyong nyong and Mayaro viruses, although substitutions are observed in the single-stranded terminal loop of the structure (Supplementary Figure S4). Within more divergent members of the Semliki Forest Complex, including Ross river virus and SFV, covariant and semi-covariant substitutions (the later involving non-canonical G:U interactions) within the base-paired stem are observed, which maintain broadly homologous structures. As has been detailed in previous studies, the length of 5′UTR regions vary greatly across the alphavirus genus (37) and more divergent alphaviruses such as VEEV, SINV or Eilat virus, lack the sequence domain within the 5′UTR responsible for SL47 formation (Supplementary Figure S4). It has previously been shown that the 5′UTR sequences of SFV and SINV are involved in controlling alphavirus template specificity for initiation of negative-strand synthesis (16). This study clearly demonstrates that SL47 is required for efficient CHIKV genome replication and that compensatory substitutions demonstrate that it functions in a structure-dependent manner within the positive-sense genomic RNA molecule, yet it is not conserved across the genus. Consequently, we speculate that SL47 may play a role in CHKV replicase template specificity, during initiation of negative-strand synthesis.

Within this study SL246 was the only RNA replication element, other than SL47, that functioned during CHIKV genome replication in mosquito derived cells. However, unlike SL47 it was not required for efficient replication in human cells. Furthermore, SL246 is much more highly conserved across divergent members of the alphavirus genera than SL47, with *in silico* UNAFOLD analysis indicating that homologous structures are capable of forming within divergent viruses such as SINV, VEEV and Eilat (Supplementary Figure S5). The conservation of this novel RNA replication element within the alphavirus genus, combined with analysis of compensatory substitutions to rescue wild-type levels of replication and mutagenesis of single-stranded regions, indicate that SL246 also functions within the positive genomic RNA molecule in a structure dependent manner during efficient genome replication.

SL165 and SL194 represent the CHIKV nsP1 51nt CSE. Although, studies investigating this element have not been previously published for CHIKV, both its RNA structure and sequence is highly conserved across the alphavirus genus and published studies in VEEV and SINV have demonstrated that this 51nt element enhances virus replication in both mosquito and mammalian derived cells, via initiation of negative strand replication (15,16,20,21). By comparison, this study demonstrates that the 51nt CSE of CHIKV acts in a host dependent manner – enhancing replication in human cells, while having no significant effect on viral replication in *Ae. albopictus* derived cells. Given the high level of conservation of the 51nt CSE, it may be hypothesised that it functions through a conserved mechanism across divergent members of the *Alphavirus* genus. However, differences demonstrated in the current study between the host specificity of the CSE in CHIKV and those in previously published studies in VEEV and SINV, suggest that the function or mechanism of action may be more divergent than previously recognized.

Following reverse genetic analysis, demonstrating that each of the nsP1 encoding region stem–loops function as RNA replication elements required for efficient genome replication, we hypothesized that the primary nucleotide sequence within single stranded bulge or terminal-loop regions may be important for mechanisms of action, for example as recognition signals for host/viral *trans* activating factors. Surprisingly, mutagenesis of these regions in SL85, SL102, SL165 and SL246 had either no significant effect or only a comparatively small effect on sub-genomic replicon or virus replication (compared to duplex-stem mutations). Combined with results demonstrating that wild-type levels of replication could be rescued in duplex-stem mutants by restoring base-pairing with further compensatory mutations, these results suggest that SL85, SL102, SL165 and SL246 function during CHIKV genome replication in a structure, rather than a primary sequence, dependent manner. In further studies we were unable to isolate infectious virus following transfection of capped RNA transcripts into Huh7 or C6/36 cells, in which all six mutant stem–loops were combined (Supplementary Figure S6). Consequently, although the exact mechanism by which the individual RNA replication elements function remains to be fully elucidated, inability to rescue virus containing multiple mutant stem–loops suggests a synergistic process - resulting in a cumulative effect, rather than functional redundancy.

In contrast to the other structures in the nsP1 encoding region, we demonstrate here that the function of SL194, as an RNA replication element during CHIKV genome replication, is dependent on both the structure of the stem–loop and the primary sequence of the single stranded terminal loop—as a single synonymous C>U substitution within this non-base paired region significantly inhibited both sub-genomic replicon and infectious virus replication (Figures 10 and 11). As this single substitution reproduced a UUUU terminal loop sequence conserved within the CSE elements of other divergent alphaviruses (including VEEV, SINV and SFV), we predicted that it would not affect CHIKV replication - the fact that replication was inhibited, suggests that the terminal loop of SL194 represents a CHIKV-specific signal motif. In addition, while the sequence or size of the terminal loop of SL165 did not significantly affect genome replication directly, virus replication as a whole was inhibited by increasing the size of the unpaired terminal-loop by four nucleotides. The primary nucleotide sequence and structure of the nsP1 CSE has previously been shown to be highly conserved between divergent alphaviruses (17). Reverse genetic evidence presented here indicates that the primary sequence of the terminal loop, rather than the structure or sequence of the base-pared stem, may contribute to CHIKV-specific functionality of this RNA replication element during the viral lifecycle.

Immediately upstream of the nsP1 51nt CSE and overlapping the AUG start codon, structure-led reverse genetic analysis demonstrated that the structure of CHIKV differs from that previously observed for other alphaviruses (17). For example, in SINV this region has been shown to form a single, long RNA element – deletion of which does not inhibit virus RNA replication (15). In the current study we demonstrate that this region of the CHIKV genome contains two RNA replication elements (SL102 and SL85), both of which are involved in efficient genome replication in human derived cells but not those from the mosquito host. Interestingly, while the *in silico* thermodynamically predicted structure, SHAPE mapping and structure-led reverse genetic analysis were in close agreement for SL102, this was not the case for SL85 (Figure 1B). Structure-led reverse genetic analysis, destabilizing and then restoring base-pairing with compensatory substitutions, clearly demonstrated that SL85 is essential for efficient CHIKV genome replication in human derived cells and functions through a structure-dependent mechanism. However, SHAPE mapping of SL85 was inconsistent with the predicted structure, suggesting that this region of the genome may be structurally dynamic and able to form alternative interactions (such as a potential pseudoknot between complimentary sequence motifs in the apical region of SL85 and an adjacent upstream region of the genome overlapping the AUG start codon) that destabilize base-pairing within the stem of SL85. While conserved in CHIKV and closely related alphaviruses such as O’nyong’nyong virus (Supplementary Figure S4), complementarity and thus potential pseudoknot formation is not conserved in more divergent members of the genus -such as SINV and VEEV. Furthermore, due to limitations in synonymous sites available for mutagenesis and the potential for disruption of the ORF-1 start codon Kozak consensus sequence, results from reverse-genetic analysis of this potential interaction remain inconclusive. Consequently, while the current study clearly demonstrates that SL85 forms and functions via a structure dependent mechanism and indicates that this is a structurally dynamic region; the structure of such an alternative interaction remains unclear. Likewise, the potential contribution of, as yet uncharacterised, RNA-RNA interactions within the negative-strand RNA intermediate remain to be investigated.

In other positive stranded RNA viruses (such as Hepatitis C virus (38–40) and flaviviruses (12,41,42)), such structurally dynamic regions have been demonstrated to act as molecular switches – in which RNA structure conformational changes, under the control of *trans* activating factors, influences essential process such switching the viral genome between mutually exclusive replication and translation modes. While the functional significance of switching between SL85 (demonstrated here as having a role in genome replication) and an alternative interaction remains unclear, work is ongoing to investigate the hypothesis that, in a similar way to dynamic initiations in other RNA viruses, it may act as a riboswitch switch during CHIKV replication.

In summary, through a structure-led reverse genetic approach, we have mapped and phenotypically analysed six RNA replication elements within the 5′UTR and adjacent nsP1 encoding region of CHIKV, demonstrating their essential role in efficient CHIKV genome replication. Furthermore, results analysing compensatory substitutions within duplex-stems and residues within unpaired regions, are consistent with these elements functioning in the positive-sense genomic transcript, through primarily structure dependent mechanisms. While elements, such as the CSE (SL165/SL194) are structurally very conserved across divergent alphaviruses, studies presented here reveal a number of novel RNA replication elements within the 5′UTR and adjacent nsP1 encoding region, specific for CHIKV and closely related alphaviruses - that we speculate may play a role in replicase template specificity during initiation of negative-strand replication. These studies are important both for our understanding of fundamental processes essential to replication of this important human pathogen and for rational design of a genetically stable attenuated vaccine.

## Supplementary Material

gkz640_Supplemental_File
