# Supplementary material for: Structural and phenotypic analysis of Chikungunya virus RNA replication elements
Source: Nucleic Acids Res. 2019 Jul 27;47(17):9296–312. doi: 10.1093/nar/gkz640 (PMC6753490; doi:10.1093/nar/gkz640)
Supplement: gkz640_Supplemental_File [file gkz640_supplemental_file.pdf]

Supplementary Table 1:

Primer sequences for reverse transcription and quantification PCRs for CHIKV RNA strand-specific quantification

CHIKV (-) strand detection	PCR	Primer sequence (5'-3')
CHIKV FT tag T	reverse transcription	GGC AGT ATC GTG AAT TCG ATG CGA CAC GGA GAC GCC AAC ATT
Tag T	quantitative	GGC AGT ATC GTG AAT TCG ATG C
CHIKV R T	quantitative	AAT AAA TCA TAA GTC TGC TCT CTG TCT ACA TGA
**CHIKV (+) strand detection**	**PCR**	**Primer sequence (5'-3')**
CHIKV RT tag T	reverse transcription	GGC AGT ATC GTG AAT TCG ATG CGT CTG CTC TCT GTC TAC ATG A
CHIKV F T	quantitative	AAT AAA TCA TAA GAC ACG GAG ACG CCA ACA TT
Tag T	quantitative	see above

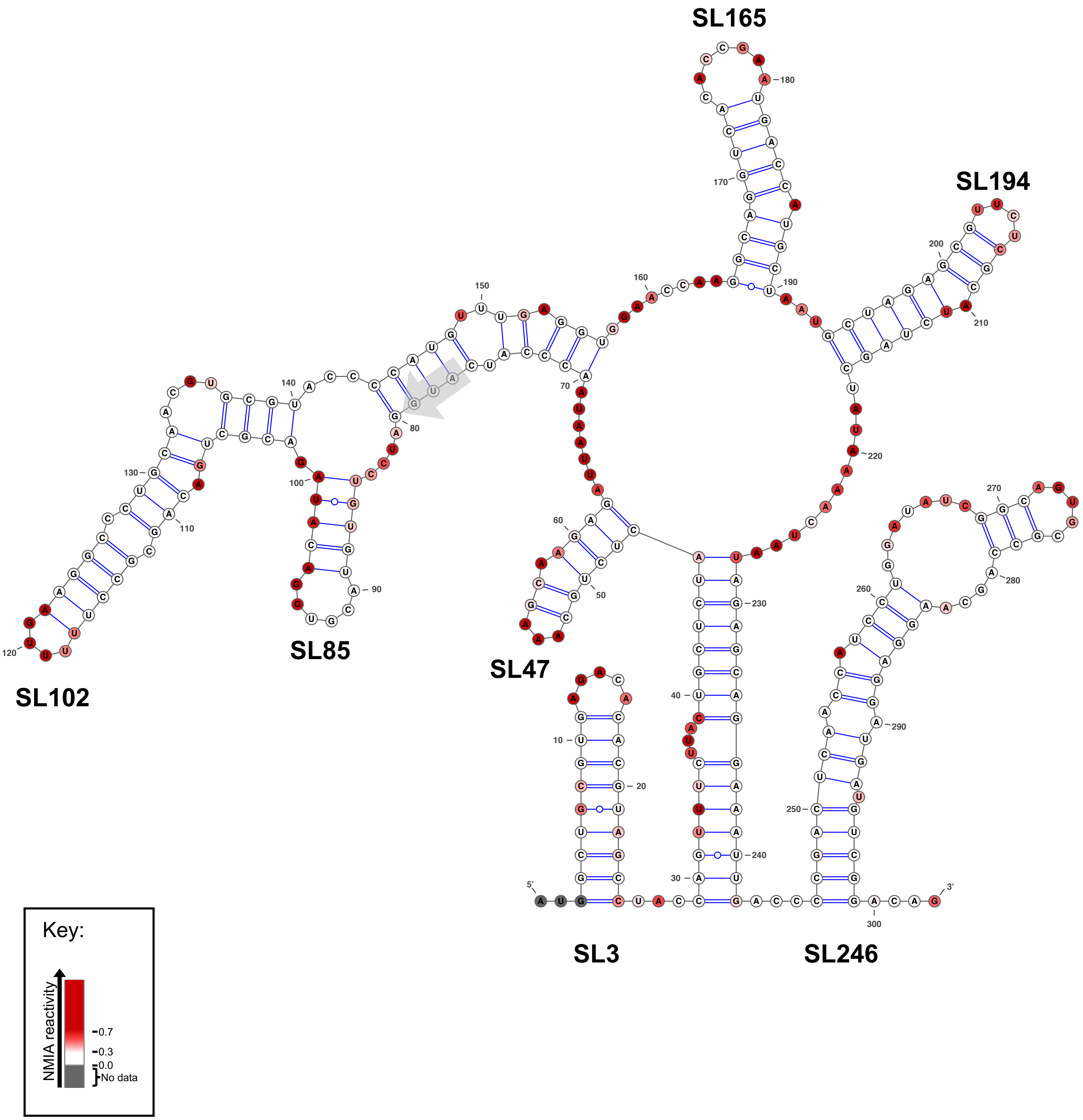

Supplementary Figure 1:

28°C SHAPE reactivities for individual nucleotides overlaid onto a 28°C thermodynamically derived model of RNA folding, generated using SHAPE-directed constraints. SHAPE reactivities are shown as a heat map: grey indicates no data, white SHAPE reactivities between 0-0.3 and increasing intensities from light pink to dark red indicate increasing SHAPE reactivities, as denoted by the key. High reactivity (red) denotes unpaired nucleotides whereas low reactivity (white) denotes base-paired nucleotides. Predicted stem-loops are labelled SL3, SL47, SL85, SL102, SL165, SL194 and SL246.

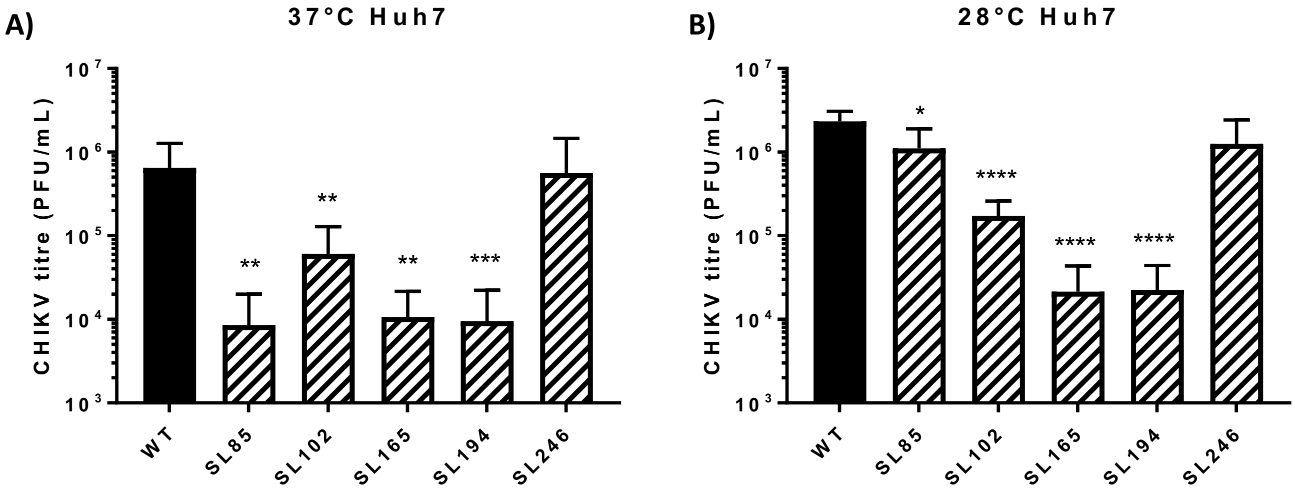

Supplementary Figure 2:

*Replication phenotype of infectious wild-type (WT) CHIKV (black bar) compared to virus bearing mutations predicted to destabilize the heteroduplex stem RNA structures (hatched bars), in Huh7 cells grown at **A)** 37°C and **B)** 28°C.*

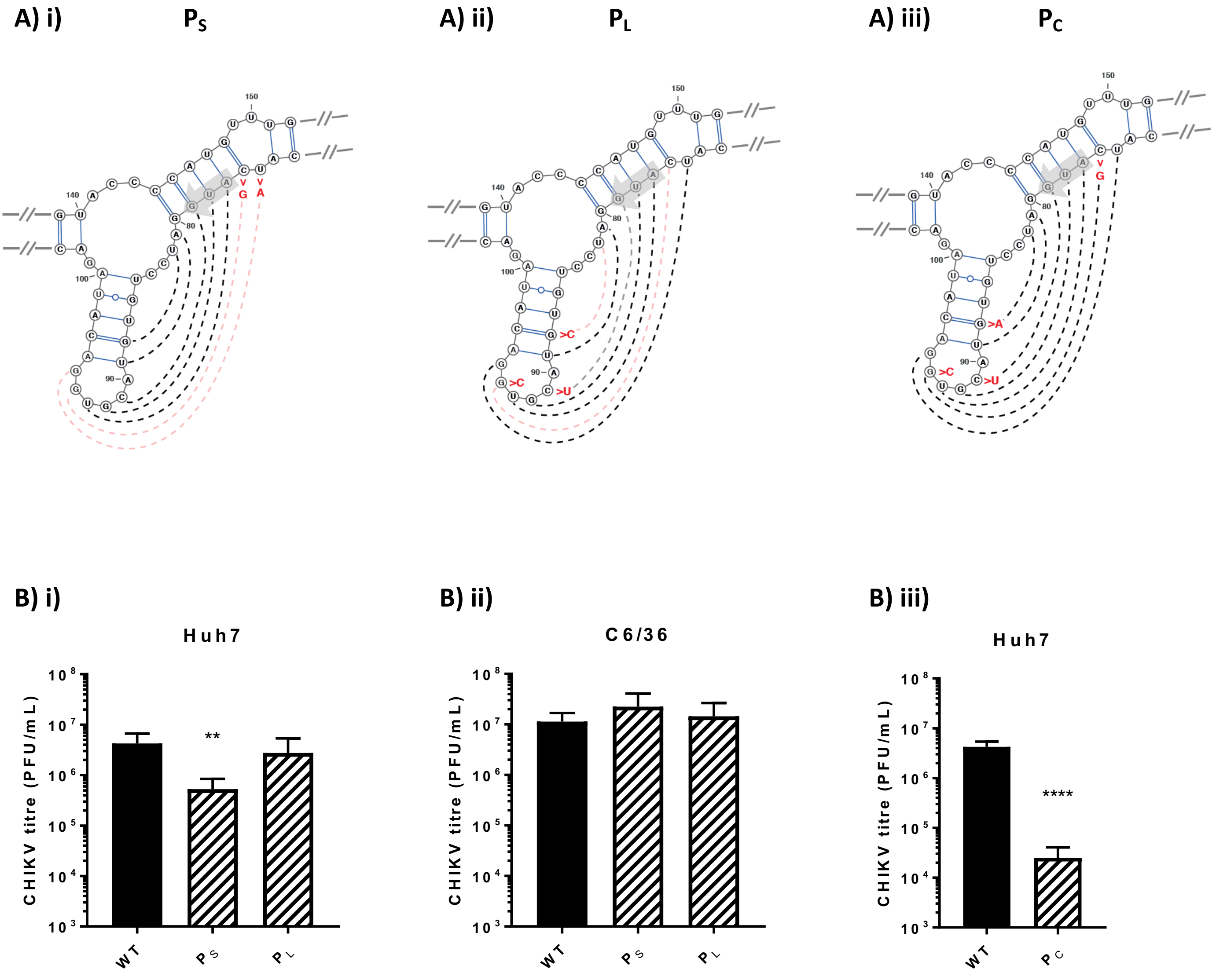

Supplementary Figure 3:

A) *Mutations predicted to destabilize the putative pseudoknot from the* ***i)*** *single-stranded (P_S) or* ***ii)*** *loop (P_L) regions (red).* ***iii)*** *A compensatory set of mutations predicted to restore base-pairing of the pseudoknot: P_C (blue). The AUG start codon of nsP1 is denoted by a grey arrow; all mutations are synonymous.* ***B)*** *Replication phenotype of infectious wild type (WT) CHIKV (black bar) compared to virus bearing mutations (hatched bars). Infections with P_s and P_L were carried out in* ***i)*** *Huh7 human and* ***ii)*** *C6/36 Ae. albopictus cells.* ***iii)*** *Huh7 cells were then infected with virus containing mutations predicted to restore base-pairing of the pseudoknot (P_C).*

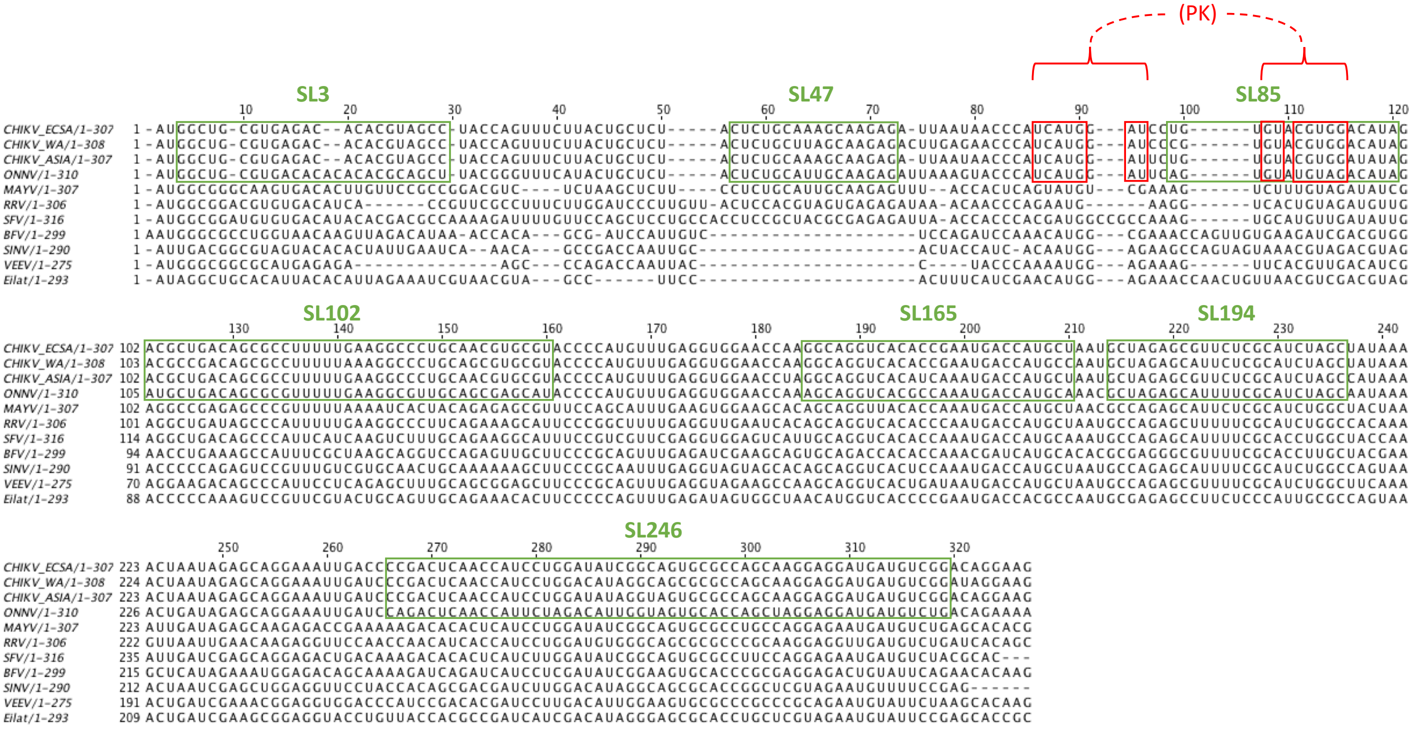

Supplementary Figure 4:

Nucleotide alignment of CHIKV ECSA, WA and Asian genotypes with other members of the Alphavirus genus - comparing the first 320nt of the genome. Predicted stem-loops for CHIKV and ONNV (green boxes) are shown, along with the putative pseudoknot structure (PK - interacting nucleotides in red boxes). Alignment gaps indicated by dashes.

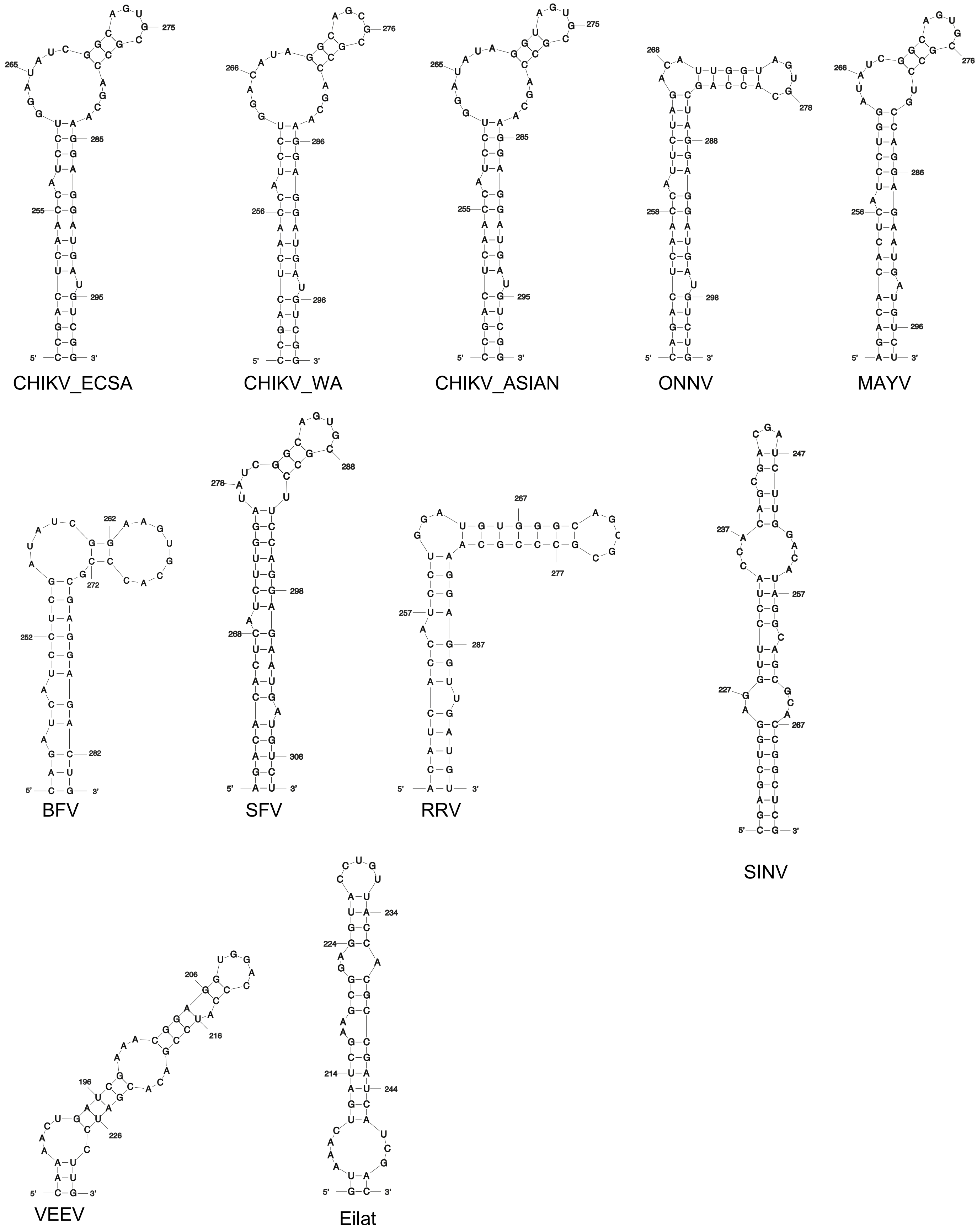

Supplementary Figure 5:

28°C in silico predicted UNAFOLD thermodynamic predictions for CHIKV SL246 homologous structures from the ECSA, WA and Asian CHIKV genotypes, closely related alphaviruses (ONNV and MAYV), other members of the complex (BFV, SFV and RRV) and from more distantly related members of the genus (SINV, VEEV and Eilat virus).

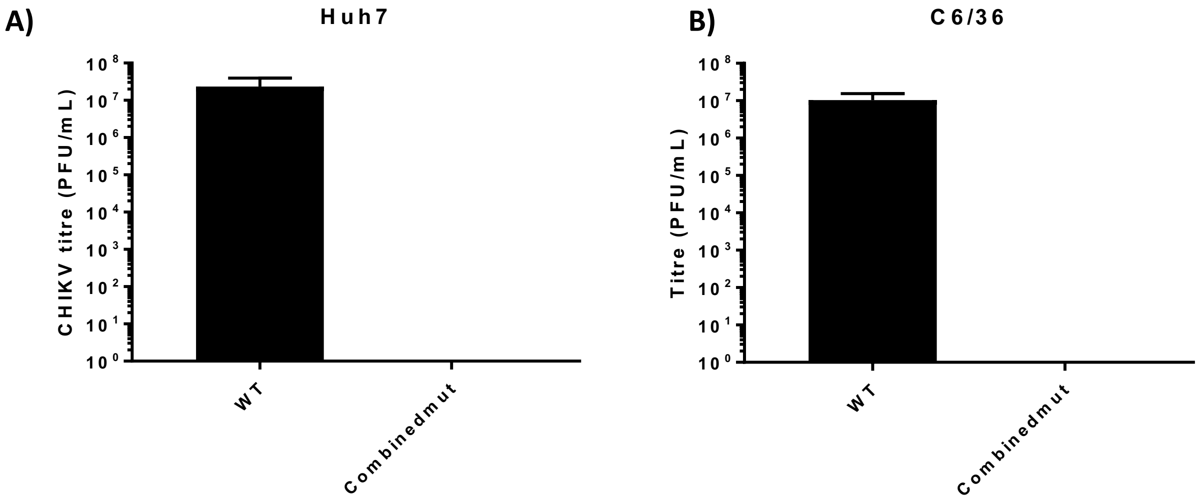

Supplementary Figure 6:

Replication phenotype of WT CHIKV (black) compared to mutant virus containing all stem-loop destabilising mutations: SL47, SL85, SL102, SL165, SL194, SL246 (denoted as Combinedmut) following harvest of supernatant 48 hours post transfection by electroporation of capped viral into ***A)*** *Huh7 cells and* ***B)*** *C6/36 cells - no combined mutant virus could be recovered in either cell line (n=3).*
